# Early Tube Feeding after Percutaneous Endoscopic Gastrostomy (PEG): An Observational Study

**DOI:** 10.3390/nu15051157

**Published:** 2023-02-25

**Authors:** Rachel Strahm, Manuel Weber, Reiner Wiest, Kai-Uwe Schmitt

**Affiliations:** 1 Department of Diabetes, Endocrinology, Nutritional Medicine and Metabolism, Bern University Hospital Inselspital and University of Bern, 3010 Bern, Switzerland; 2 Academic-Practice-Partnership of Bern University of Applied Sciences and Insel Gruppe (Bern University Hospital Inselspital), 3008 Bern, Switzerland; 3 Department of Visceral Surgery and Medicine, Bern University Hospital Inselspital, 3010 Bern, Switzerland

**Keywords:** percutaneous endoscopic gastrostomy, artificial nutrition, tube feeding, enteral nutrition

## Abstract

This study investigated whether enteral nutrition by early tube feeding led to changes in clinical parameters compared to tube feeding after 24 h. Starting on 1 January 2021, and following the latest update of the ESPEN guidelines on enteral nutrition, patients with percutaneous endoscopic gastrostomy (PEG) received tube feeding 4 h after tube insertion. An observational study was conducted to analyze whether the new scheme affected patient complaints, complications, or hospitalization duration compared to the previous procedure of tube feeding starting after 24 h. Clinical patient records from one year before and one year after the introduction of the new scheme were examined. A total of 98 patients were included, and of those 47 received tube feeding 24 h after tube insertion, and 51 received tube feeding 4 h after tube insertion. The new scheme did not influence the frequency or severity of patient complaints or complications related to tube feeding (all *p*-values > 0.05). However, the study showed that the length of stay in hospital was significantly shorter when following the new scheme (*p* = 0.030). In this observational cohort study an earlier start of tube feeding did not produce any negative consequences but did reduce the duration of hospitalization. Therefore, an early start, as suggested in the recent ESPEN guidelines, is supported and recommended.

## 1. Introduction

Disease-associated malnutrition is a common problem in hospitals, showing a prevalence of 20–60% and a negative impact on morbidity and mortality rates [1]. If patients are unable to eat for more than 1 week or if their energy and protein intake is below 60% of the required amount for at least 1–2 weeks, artificial nutrition is indicated [2,3]. Enteral nutrition can be administered through a transnasal or a percutaneous tube [4]. Depending on the nutritional status and the underlying disease, the duration of enteral nutrition must be estimated in advance. If enteral nutrition is expected to be required for a prolonged period, a percutaneous endoscopic gastrostomy (PEG) is usually inserted [2]. Indications for a percutaneous tube include dysphagia due to neurological diseases, swallowing difficulties and/or stenosing processes due to a tumour or caused by trauma [5,6]. The complication rate after PEG placement is estimated to be 8–30% [7], but the definition of complications varies widely. Serious complications occur in approximately 1–4% of cases, while acute and severe complications, such as perforation, severe peritonitis, or serious haemorrhage requiring surgical intervention, are observed in less than 0.5% of cases [7]. The most common complications after PEG placement include wound infection, hypergranulation, skin irritation, haemorrhage, leakage and tube dislocation [2,6,7,8].

Generally, the PEG is inserted by a gastroenterologist. Prior to 2021, a typical procedure required patients to fast for at least 8 h before PEG placement and usually for 6 h afterwards, and then artificial feeding commenced 24 h after insertion. The first dressing change was performed within the first 24 h by a nurse together with the gastroenterologist to check the protective dressing, with a focus on bleeding signs. Starting on 1 January 2021, the Department of Gastroenterology of Bern University Hospital adopted procedures according to the updated 2020 guidelines of the European Society for Clinical Nutrition and Metabolism (ESPEN), i.e. the feeding scheme for enteral nutrition was changed from 24 h to 4 h after PEG placement. 

The objective of this study was to analyze the impact of changing the feeding scheme from 24 h to 4 h after insertion of a percutaneous endoscopic gastrostomy (PEG) with respect to clinical parameters, such as patient complaints or complications. It was hypothesized that the new scheme would not result in more complications.

## 2. Materials and Methods

The potential consequences of early enteral nutrition after insertion of a percutaneous endoscopic gastrostomy (PEG) were investigated in a pre–post observational cohort study; the study relates to quality of care. All data were retrieved from the hospital’s electronic patient records. One group was represented by patients who received a PEG under the updated scheme (i.e. tube feeding after 4 h). It was decided to consider one entire year of the new scheme, from 1 January to 31 December 2021. The comparison group (feeding after 24 h) covered one year prior to the implementation of the early feeding scheme, i.e., from 1 January to 31 December 2020.

### 2.1. Inclusion and Exclusion Criteria

Only patients who received a PEG (Freka^®^ PEG tube CH 15) or Pexact (Freka^®^ Pexact tube CH 15) at the Department of Gastroenterology of Bern University Hospital were included. Inclusion criterion: patients aged 18 years and older. Exclusion criteria any of the following: (i) tubes inserted solely prophylactically, (ii) for drainage, (iii) exclusively for the administration of medication and water, PEG-insertion at the intensive care unit, (iv) if patients were transferred directly to another hospital after PEG placement, (v) if there was a lack of access to patient documentation, or (vi) if general consent was denied.

### 2.2. Data

Every PEG insertion by a gastroenterologist at the Department of Gastroenterology of Bern University Hospital between 1 January 2020 and 31 December 2021 was checked and filtered according to the inclusion/exclusion criteria. All data were extracted from the digital hospital documentation system and pseudonymized by two research assistants. In addition, data of patients with complications were examined and categorized by gastroenterologists. Complications were categorized based on the patient records with respect to reported pain (no pain/ mild pain/ moderate or severe pain), related to tube feeding (yes/no), and concerning complications after tube insertion (no symptoms/ mild symptoms/ moderate or severe symptoms). The latter type of complications was classified by a gastroenterologist in line with the examples provided in the introduction above. 

Data included sociodemographic information, hospitalization duration, underlying primary diagnosis, indication for tube placement, type of previous oral nutrition, type of tube, type of tube feeding application, complaints directly related to tube feeding and tube insertion, and complications directly related to tube insertion. Data was extracted in Excel (Microsoft^®^).

### 2.3. Statistical Analysis

All responses were included in the analysis regardless of missing data. Descriptive and inferential statistics were used for analyses. Chi-Square tests were performed to determine if there were significant differences in proportions between the two groups. Since the data for the variables age, body mass index and duration of hospitalization were not normally distributed in at least one of the two groups (Shapiro-Wilk tests; *p* ≤ 0.001), Mann-Whitney U tests were conducted to compare the two groups. The significance level was set at *α* = 0.05 (2-sided). Analyses were conducted using IBM SPSS Statistics for Windows (Version 28.0. IBM Corp., Armonk, NY, USA)

## 3. Results

A total of 47 patients who had received a percutaneous endoscopic gastrostomy and who met the eligibility criteria for the early feeding condition were identified in the clinical documentation system. For the retrospective group, 51 patients who had received enteral tube feeding after 24 h were included. The two groups and the outcomes are summarized in Table 1. 

The two groups did not differ significantly in their socio-demographics (all *p*-values > 0.05). The sample included mainly patients from oncology. Dysphagia was the dominant reason for tube feeding. The data showed that the use of PEXACT increased significantly, from 22% to 51%, after implementing the new scheme (*p* = 0.002). The change of the procedure resulted in a significant shift from bolus feeding to continuous feeding (*p* = 0.026). While 45–50% of the patients experienced mild pain related to the tube, less than 10% reported moderate to severe pain. Complaints or symptoms related to tube feeding increased slightly with the new procedure, from 16% to 23%, but statistically were not significant. Severe complications after tube insertion were slightly reduced but were still very low in both groups. The statistical analysis revealed that the entire length of stay at hospital (*p* = 0.030) and the time span from tube insertion to discharge from hospital (*p* = 0.027) were significantly lower in patients following the new feeding scheme.

## 4. Discussion

Changing the feeding scheme has the potential to influence quality of care. While the updated ESPEN guidelines recommend early tube feeding after insertion of a percutaneous endoscopic gastrostomy (PEG) [3], there was a lack of studies reporting the impact of switching the feeding scheme. Generally, the recommendation to start feeding early is not new and some studies have already suggested that early feeding after PEG insertion is safe and not associated with an increase of complications or adverse events [9,10]. A meta-analysis by Szary et al. (2011) [11], however, showed that an early artificial feeding scheme is often implemented reluctantly due to a lack of established guidelines or procedures, concerns about complications, or unwillingness to alter existing habits. Studies are thus needed that address the impact of changing clinical procedures. This study bridges this gap in that it contributes to an evidence-based clinical practice and quality assurance.

Our study demonstrated that there was no significant difference in complications after tube insertion with early versus late tube feeding. According to the literature, serious complications occur in about 1–4% of cases [7]. In our study, severe complications like pneumoperitoneum with peritonitis (*n* = 1), aspiration pneumonia (*n* = 1) and bleeding with leakage (*n* = 1) occurred in 2.1–3.9% of the cases, i.e. they were within the known range. Wesley et al. (2021) [9] investigated the tolerability of artificial feeding after PEG insertion at different times (after 12, 24 and 48 h) without noting a significant difference for feeding intolerance and earlier beginning with artificial nutrition. This work is in line with this finding in that complaints related to tube feeding were only slightly, but not significantly, higher in the group with fast tube feeding. 

In the group with early feeding, PEXACT tubes were inserted significantly more often than PEG tubes. This can be explained by an adaptation of the practice at the department of the gastroenterology based on findings that for some oncological patients the risk of tumor spreading is reduced by not pulling the plate through the tumor [12]. There was also an increase in continuous feeding over bolus feeding. Reasons for this change cannot fully be explained because the nutritional recommendations were not changed. The administration of artificial nutrition can be by boli or continuously. How to applicate the nutrition should be discussed in a multidisciplinary team together with the patient whereby the patient’s disease, feed tolerance and patient preference are factors that should be considered [2]. 

The length of stay in hospital was significantly shorter when patients were artificially fed earlier. This can possibly be attributed to the fact that artificial feeding can be started on the same day as tube insertion. From a nutritional perspective, an early start shortens the fasting time of patients. Hence, the administration and tolerability of enteral nutrition can be checked earlier and adjusted, if necessary. This is particularly relevant if discharge from hospital is planned for the day after tube insertion. Furthermore, if there is a risk of refeeding syndrome, monitoring and possible therapy can be initiated earlier. Thus, the earlier start of tube feeding can positively influence clinical processes resulting in an earlier discharge.

## 5. Conclusions

This study analyzed the effect of starting enteral feeding within 4 h after tube placement, as recommended by the updated 2020 ESPEN guidelines. Investigating the potential impact of a change in guidelines contributes to evidence-based practice in clinical nutrition and is thus relevant in the context of clinical quality and patient safety. The study found no negative consequences of an earlier start of tube feeding. There was no indication that the new procedure influenced the frequency or severity of patient complaints or complications related to tube feeding. However, the study found that the length of stay in hospital was significantly reduced when following the new scheme. This indicates that changing the feeding scheme after insertion of a percutaneous endoscopic gastrostomy (PEG) can have a positive impact on clinical procedures resulting in an earlier discharge. Consequently, it is recommended to implement the updated ESPEN guidelines in clinical practice.

## Figures and Tables

**Table 1:** Sample characteristics and results of the statistical comparison (*N* = 98).

	Tube Feeding after 24 h	Tube Feeding after 4 h	*p*-Value
**Sample size** [*n*]	51	47	
**Socio-demographics**			
Age [years] (M, Min, Max)	65, 31, 89	65, 18, 86	0.873
Female (*n*, %)	19, 37.3	19, 40.4	
Male (*n*, %)	32, 62.7	28, 59.6	0.748
Body Mass Index [kg/m^2^] (M, Min, Max)	22.9, 14.0, 41.3	22.1, 11.6, 31.3	0.839
**Hospitalization**			
Entire length of stay [days] (M, Min, Max)	27.3, 2.0, 111.0	15.4, 2.0, 127.0	0.030
Days from tube insertion to discharge [days] (M, Min, Max)	15.0, 2.0, 70.0	9.5, 2.0, 88.0	0.027
**Pathophysiological classification (primary mechanism)**			
Oncological (*n*, %)	27, 52.9	30, 63.8	
Neurological (*n*, %)	19, 37.3	17, 36.2	
Trauma (*n*, %)	4, 7.8	0, 0.0	
Other (*n*, %)	1, 2.0	0, 0.0	0.164
**Indication for tube insertion/feeding**			
Dysphagia (*n*, %)	36, 70.6	34, 72.3	
Oral abstention (*n*, %)	12, 23.5	10, 21.3	
Anorexia (*n*, %)	2, 3.9	2, 4.3	
Assumed progressive deterioration in oral intake (*n*, %)	1, 2.0	1, 2.1	0.995
**Type of oral nutrition**			
Regular diet (*n*, %)	5, 9.8	2, 4.3	
Soft or fluid food (*n*, %)	31, 60.8	34, 72.3	
Fasting, no oral intake (*n*, %)	15, 29.4	11, 23.4	0.391
**Type of tube**			
PEG (*n*, %)	40, 78.4	23, 48.9	
PEXACT (*n*, %)	11, 21.6	24, 51.1	0.002
**Type of feeding application**			
Bolus (*n*, %)	31, 60.8	18, 38.3	
Continuous (*n*, %)	20, 39.2	29, 61.7	0.026
**Complaints/symptoms directly related to tube itself**			
Moderate to severe pain (*n*, %)	5, 9.8	3, 6.4	
Mild pain (*n*, %)	23, 45.1	23, 48.9	
No pain (*n*, %)	20, 39.2	21, 44.7	
Unknown/NA (*n*, %)	3, 5.9	0, 0.0	0.773
**Complaints/symptoms directly related to tube feeding**			
Yes (*n*, %)	8, 15.7	11, 23.4	
No (*n*, %)	43, 84.3	36, 76.6	0.334
**Complications after tube insertion**			
Severe complications (*n*, %)	2, 3.9	1, 2.1	
Mild complications (*n*, %)	18, 35.3	17, 36.2	
No complications (*n*, %)	31, 60.8	29, 61.7	0.876

Notes. h: h; M: mean; Min: minimum; Max: maximum; NA: not available.

## Data Availability

The datasets generated and analyzed during the current study are available from the corresponding author upon reasonable request.
